# Translucency of a Dental Porcelain Mixed by Two Ceramic Slurry Methods: A Bayesian Comparison

**DOI:** 10.1155/2022/6666931

**Published:** 2022-06-06

**Authors:** Catalina Serna-Meneses, Gabriel Ocampo-Parra, Santiago Arango-Santander, Claudia Garcia-Garcia, Luis Felipe Restrepo-Tamayo, Johnatan Cardona-Jimenez, Alexander Ossa, Alejandro Pelaez-Vargas

**Affiliations:** ^1^Universidad Cooperativa de Colombia, Medellín, Colombia; ^2^School of Physics, Universidad Nacional de Colombia, Medellín, Colombia; ^3^Private Practice in Prosthodontics, Medellín, Colombia; ^4^Universidade de São Paulo, Institute of Mathematics and Statistics IME-USP, São Paulo, Brazil; ^5^Facultad de Ingeniería, Institución Universitaria Pascual Bravo, Medellín, Colombia; ^6^Production Engineering Department, Eafit University, Medellín, Colombia

## Abstract

**Background:**

The ceramics industry produces porcelain pastes using a controlled ratio of water and porcelain powder. Two methods are used to produce a dental porcelain paste: one-step mixing or incremental mixing.

**Objective:**

To evaluate the optical properties of a feldspathic dental ceramic using two different ceramic paste preparation methods using a Bayesian approach.

**Materials and Methods:**

Two groups of feldspathic porcelain discs, an incremental mixing group (*n* = 40) and a one-step mixing group (*n* = 40), were assessed. Groups were evaluated using spectrophotometry, and the translucency parameter (TP) of each sample was calculated. Surfaces were characterized by AFM and SEM. Statistical analysis was performed using a Bayesian approach.

**Results:**

Translucency parameter values of the incremental mixing group ranged from 1.65 to 3.41, while values for the one-step mixing group ranged from 3.62 to 5.74, this difference being statistically significant. The lowest roughness was obtained on the surface of discs in the one-step mixing group.

**Conclusions:**

Feldspathic porcelain with lower translucency and higher roughness was obtained using the incremental mixture method.

## 1. Introduction

Feldspathic dental porcelains are composed by an amorphous matrix (K_2_O-Al_2_O_3_-SiO_2_) with a dispersion of leucite particles and pigments obtained from metal oxides. These oxides are responsible for the reduced melting temperature of the material, and they determine the color and opacity [1]. Low-melting point porcelains used in fixed dental prostheses have desirable properties, such as excellent aesthetics, high biocompatibility, wear strength, and highly stable chemical components, which make them the material of choice to match the natural teeth. When matching a porcelain crown with the natural teeth, the size, shape, surface texture [2], opalescence, and translucency of the material are important to consider [3]. Porcelain restorations reproduce translucency and the color of natural teeth for aesthetic reasons [4], but clinical results may also be the result of human factors, such as proper communication between the dental professional and the technician or factors from the natural substrate [5, 6]. This communication has been widely studied as highly relevant to achieve better results, but persistent trends show that flawed communication produces low satisfaction [6].

Translucency is the relative amount of light transmission or diffuse reflectance from the surface of a substrate through a semiopaque medium [7]. Translucency may be affected by many factors [8], including thickness [9, 10], microstructure [11], roughness [12], and the number of ceramic staining and firing cycles [13], all of which affect light scattering. If most of the light passing through a ceramic material is highly scattered and diffused, the material appears opaque. If only a part of the light is scattered and most of it is diffusely transmitted, the material will appear translucent. The amount of light that is absorbed, reflected, and transmitted depends on the concentration of crystals within the core matrix, the chemical composition of the crystals, and the particle size, all of which affect the wavelength of the light reflected and transmitted. Wavelength-size particles produce greater dispersion. Both the chemical composition of the particles (which also affects absorption) and the degree of refraction of the particles in the matrix affect the level of scattering [10]. High numbers in translucence parameters are reported with thin layers of veneer ceramics [14].

Translucency has been evaluated for different ceramic systems grouped by the processing method, such as condensation, hot pressing, casting, computer-aided milling, or machining [3, 12, 15–29], with a wide interval in the total thickness of specimens ranging from 0.3 mm to 2.3 mm. Such values might not be clinically relevant.

Preparation of dental porcelain involves several steps that must be carefully controlled to ensure the quality of final restoration. The process of producing high-quality dental porcelain powder involves controlling the particle size and chemical composition. Further processing by the dental technician and the dentist includes mixing the ceramic paste, shaping, sintering, and polishing of dental restorations [30]. Addition of defects during fabrication of porcelain restorations adversely affects optical properties.

In the ceramics industry, water is mixed with a porcelain powder in a very controlled manner to produce a porcelain paste. These slurries are manufactured using methods that include slip casting (filtration), electrophoresis, tape casting (evaporation), extrusion, and injection molding. In dentistry, the porcelain paste is similarly prepared using different methods with different objectives and outcomes. The method of mixing distilled water with the porcelain powder to reduce the presence of agglomerates during the consolidation of dry powers has already been explained for industrial scales. The second method, being an empirical approach, has been used in dental laboratories to achieve a good paste consistency that may result in intra- and interoperator variability [3, 31, 32].

The former method, known as the incremental technique, consists in adding distilled water in small increments to the powder, while the latter, known as the one-step method, consists in adding a predetermined amount of distilled water to the ceramic powder at once. The one-step method may be performed in a controlled manner to obtain a ceramic paste, as it was already explained. However, manually sculpting/building-up might require a higher skilled technician.

The objective with both methods is to obtain a porcelain slurry with an optimal powder/liquid ratio (∼2.8 g/ml), which yields a creamy consistency [3, 32]. In this mixture, the liquid occupies small spaces between the ceramic particles, acting as a lubricant for the movement of these particles and producing a highly-dense green state in the final firing of the ceramic [3, 32–37], which presents homogeneously distributed microdefects and uniform porosity [38, 39]. Excess water in the porcelain slurry evaporates during sintering and may result in higher porosity. Moreover, excess water may react with some ions existing in porcelain, thus inducing changes in the microstructure and the relative amounts of crystalline and glassy phases [40, 41].

Currently, there are two approaches to determine the color and translucency of dental ceramics [42, 43]. The instrumental approach is based on spectrophotometers, colorimeters, digital cameras with specialized software [44, 45], or combinations of these systems. Spectrophotometers have demonstrated to be useful in dental research because they measure the optical coherence of opaque and translucent objects [20, 46]. Lim et al. [18] compared the translucency of eleven ceramic core materials using a spectroradiometer and a spectrophotometer. Their results showed differences when comparing both technologies, but translucency values were highly correlated. Ahn and Lee [20] found differences in the translucency of porcelain using different light sources, such as incandescent or fluorescent lamps. Digital images obtained from CCD sensors coupled to different devices (i.e., dedicated image acquisition systems, cameras, or smartphones) may be used to acquire images with several types of illumination (from 3000 k to 7000 k) and background (black and white) to correct the environmental effects. However, this method is time-consuming because it needs (1) image digital postprocessing using generic commercial software (image by image) or (2) to create a new code or written routines using available libraries on commercial or free programming computing platforms. More recently, a new generation of spectrophotometers coupled to image acquisition systems have become available for clinical use and are based on the fact that better communication between the professional and the dental technician is mandatory. However, 3D intraoral scanners, which are a part of new 3D digital dental flow systems, also allow color selection from color 3D models. To the best of our knowledge, scanners do not exactly match spectrophotometers in determining the tooth color, and additional methods are recommended, even though they have >85% repeatability [47].

The second approach is the visual method, the most widely used in dentistry, where color is matched using tooth-form shade tabs. Evans et al. [48] found significant color differences between the two dental porcelains prepared by different condensation methods when a subjective visual evaluation was conducted. The comparison between instrumental and visual approaches for color evaluation has been widely studied in dentistry.

Bayesian statistics comprises three elements, namely, prior distribution, likelihood, and posterior distribution [49]. Prior distribution reflects the initial knowledge of the event studied. This distribution may be noninformative (when all possible values for the event are equally likely) or informative (some possible values for the event are strongly, vaguely, or substantially likely); this information might be obtained from clinical, reference, skeptical, and enthusiastic priors [49]. Likelihood is obtained from experimental data. Finally, posterior distribution is a combination of the first two elements, where the prior knowledge is updated with experimental values. Bayesian statistics differs from classical or frequentist statistics in certain aspects; specifically, the concept of probability in Bayesian statistics is based on the principle of uncertainty rather than variability, which is the essential feature of classical statistics [49]. Bayesian statistics is exact, whereas classical statistics is asymptotic. Hence, the Bayesian approach does not require large sample sizes or the repetition of random experiments, as is the case in the classical statistics approach.

The objective of this study was to evaluate the optical properties of a feldspathic dental ceramic using a Bayesian approach after two different ceramic paste preparation methods were performed.

## 2. Materials and Methods

Low-melting-point dental porcelain (VitaVM13 Dentine, shade 3M2, Vita Zahnfabrik, Germany) was used in the present study. This porcelain was used to prepare discs of 13 mm in diameter and 1.5 mm in thickness [3, 23, 50]. These samples were divided into two groups, which were prepared using one of the following two methods [51]: incremental mixing, in which the discs were prepared from a paste produced by incrementally adding distilled water to the porcelain powder with a laboratory brush (#8, Kolinsky, Renfert, Germany); and one-step mixing, in which the discs were prepared from a paste produced by a single addition of distilled water to the porcelain powder in a ratio of 1 g of powder per 0.4 ml of distilled water [3, 31].

All discs were prepared using a 15 mm-diameter plastic syringe (Becton Dickinson and Co., Franklin Lakes, NJ). The syringe tip was sectioned, polished and used as the base for the discs [52]. In both methods, the ceramic paste was prepared at room temperature on a glass tile and then loaded into the syringe with a brush. The porcelain was condensed with an absorbent paper towel to remove excess water [52]. The syringe plunger was then placed at 2 mm from the top using a periodontal probe.

The surface of the disc was leveled on a flat surface to ensure uniform thickness before removing the disc from the syringe [3]. Samples were removed in the green state with a scalpel and placed on a refractory platform (Vita Zahnfabrik). All discs were heat-treated in a furnace (Vacumat 40, Vita Zahnfabrik) according to the manufacturer (temppredrying = 500°C; timepredrying = 6 min; tempslope = 55°C/min; tempmax = 880°C; timemax_temp = 1 min; and timeVAC = 7.05 min). After sintering, the surfaces of discs were polished with a series of SiC grinding papers (80–600 grit, Abracol, Colombia) using a polishing machine (LaboPol-5, Struers, Denmark) [37]. For standardization purposes, preparation of all disc specimens was performed by the same operator.

Translucence measurements were made using a spectrophotometer (Ocean Optics PC2000, USA) at wavelengths between 400 and 800 nm, which produced values in the CIELab color scale [53–56]. Illumination from the light source was daylight (D65). The translucency parameter (TP) was calculated 5 times at the center of the disc in each disc sample [57], and the difference in color of a sample when measured against black and white backgrounds was included. The TP value is zero when the material is completely opaque, higher values indicate greater translucency. This parameter was calculated using the following expression [20]:

(1) $$\begin{array}{c} y_{ij}=\left(\mu +\;\tau_{i}\right)+\;\varepsilon_{ij}, \end{array}$$

where the subscripts *W* and *B* correspond to white and black backgrounds, respectively. In this expression, *L* indicates brightness (*L* = 0 represents black and *L* = 100 white), *a* indicates the level of red or green (negative values of *a* indicate green and positive values indicate red), and *b* indicates the level of yellow or blue (negative values of *b* indicate blue and positive values indicate yellow).

Surface topography was evaluated using atomic force microscopy (Nanosurf Easyscan 2, Switzerland) in the contact mode. AFM images were acquired after the sintering cycle. Roughness was calculated in terms of amplitude parameters using a free software (Gwyddion, v. 2.59) [58]. Four boxes of 5 × 5 *μ*m per image were analyzed.

A Bayesian approach was used to calculate the sample size and to compare TP obtained with both ceramic mixture methods. In order to determine the optimal sample size, the “SampleSizeMeans” function in the R software was used. This algorithm uses a strictly Bayesian approach. It was assumed that the measurements were independent and followed approximately normal distribution. Initial samples of 10 discs produced with each of the mixing procedures were used as *a priori* information to determine the sample size.

Bayesian variance analysis was used to compare the results of the two methods.

The model follows the following equation:

(2) $$\begin{array}{c} y_{ij}=\left(\mu +\;\tau_{i}\right)+\;\varepsilon_{ij}, \end{array}$$

where *y*_*ij*_ represents TP (translucency parameter) as a function of prepared discs (*j*=1  to 80) and method used (*i*=1,2). *μ* represents the overall mean of the translucency parameter, *τ*_*i*_ (*i* =1, 2) represents the effect of the preparation method, and *ε*_*ij*_ represents the error as normal distribution (0, *σ*^2^). Prior distributions were defined as normal distribution for *μ* (0, 10^6^) and *τ*_*i*_ (0,  10) These prior distributions were defined as “slightly” informative, so inference results were obtained solely from experimental data. To perform inferential analysis, results were analyzed in the programming environment R [59] using the “rjags” library [60].

## 3. Results

**Figure 1:**
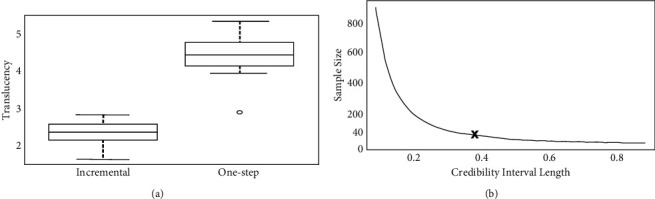
Results for the pilot sample using both groups (a) and sensitivity plot for the optimal sample (b).

Figure 1 shows the differences in TP values of discs (*n* = 10) produced for both ceramic mixture methods and used to calculate prior distribution.  Figure 1(a) shows that the minimum difference between both mixture methods was ≤1.13. Assuming this value, and resources available for experimentation, the optimal sample size was set at 40 discs for each ceramic mixture method.  Figure 1(b) shows 0.40 as the maximum length in the posterior credibility interval.

Table 1 shows 95% highest probability intervals for the mean TPs of discs produced using the two methods under study. For both statistical approaches, the incremental mixture method showed lower TP values compared to the one-step mixture method.

**Figure 2:**
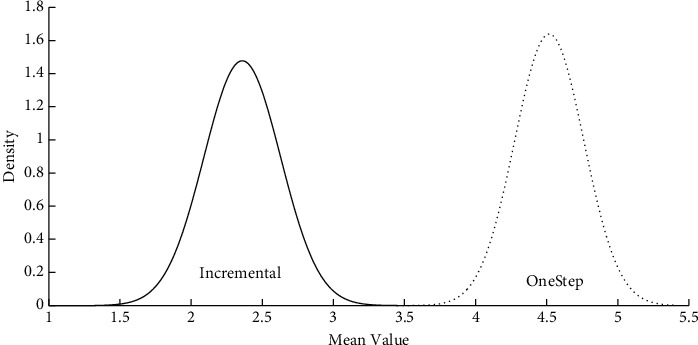
Posterior distribution for the overall mean TP (*μ*) in the highest probability intervals and the mean TPs (*μ*_*i*_) for incremental (A  *μ*_1_) and one-step (B  *μ*_2_) mixing methods.

Figure 2 shows TP posterior distribution for both mixing methods. It may be inferred that the mean TP for the incremental group ( *μ*_1_=2.45) is statistically lower than that of the one-step group ( *μ*_2_=4.49).

**Figure 3:**
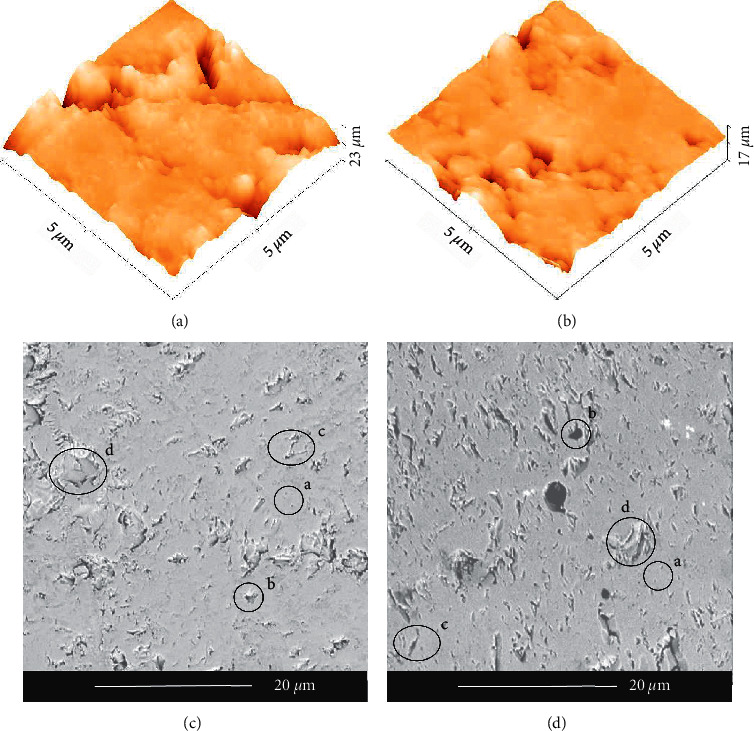
SEM and 3D AFM topographic images of discs prepared by incremental (right column) and one-step mixture (left column) methods. Fine close porosity (a), coarse close porosity (b), open porosity (c), and interparticle porosity (d).

3D AFM images (Figure 3) obtained from discs produced by incremental (*S*_*a*_ = 44.0 (11.3), *S*_*q*_ = 60.9 (15.3)) and one-step (*S*_*a*_ = 29.5 (5.4), *S*_*q*_ = 42.7 (8.1)) methods revealed statistically significant differences in roughness evaluated in terms of the two first moments of height distribution (*S*_*a*_*, S*_*q*_). Tendency of third and fourth moments of height distribution (Ssk and excess kurtosis) for both groups showed negative values for the skewness, meaning that the left tail is long relative to the right tail of distribution, and positive values for excess kurtosis, meaning that there is a “heavy-tailed” distribution for both groups.

Imperceptible morphological differences were observed in SEM analysis between both preparation methods (Figure 3). Fine and coarse close microporosity, open microporosity, and interparticle porosity were found in discs from both preparation methods. Fine and close microporosity may be responsible for the optical reflectance of the surface, while open and interparticle porosity may reduce the mechanical behavior since they are considered stress concentrators that induce fracture.

## 4. Discussion

The Bayesian approach used in the present study yielded a final sample size of 40 discs per technique, which was a significantly larger sample size than that used in other studies of optical properties of dental ceramics. Ahn and Lee [20] measured the differences in translucency of various ceramics under different light sources using a sample size of 7 discs per ceramic. Wang et al. [27] studied the translucency of various thicknesses of dental porcelain using a sample of 6 discs per thickness and 5 for zirconia. Lim et al. [18] used a sample of 7 discs to measure the translucency of ceramic materials with a spectroradiometer and a spectrophotometer. In the evaluation of mechanical properties, the sample sizes of these studies were similar to those of the present study. Pelaez-Vargas et al. [31] used a sample size of 50 discs from each technique to evaluate biaxial flexural strength with the same two methods used for porcelain paste preparation. Fleming et al. [32] evaluated the influence of the variability induced by the porcelain paste mixture on biaxial flexural strength using a sample of 30 discs.

The goal of this study was to determine the effects of the ceramic paste preparation method on the optical properties of dental porcelain. Significant differences in the TP of the discs prepared with the two methods were observed. TP obtained with the incremental mixing method was significantly lower than that obtained with the one-step method. The lowest TP (1.65) was obtained with the incremental mixing method, indicating higher opacity. The highest TP (5.74) was obtained with the one-step method, showing that this technique provides greater translucency.

The lower TP resulting from the incremental mixing technique may be explained by the smaller number of pores existing in the porcelain structure when compared with the one-step method. Pelaez-Vargas et al. [31] showed that the amount of liquid contained in green one-step samples varied with relative humidity. Higher porosity is expected in these samples because there is more space between the particles after water evaporation and reduction in the amount of water during sintering results in higher porosity [32, 39]. Zhang et al. [3] studied the influence of the powder/liquid ratio on the porosity and translucency of dental porcelains and found that porosity was susceptible to the powder/liquid ratio, while translucency was not. Another possible explanation for low TP values of the samples prepared with the incremental mixing method is the low amount of the amorphous phase in these samples, as observed by X-ray diffraction (XRD) analysis by Pelaez-Vargas et al. [31]. The amorphous phase is responsible for the optical properties of dental porcelains. A low percentage of amorphous phase indicates that other phases, such as the leucite phase, are more abundant, which may reduce translucency [41]. Ahn and Lee [20] observed differences in translucency of various ceramics exposed to different light sources using a spectrophotometer. These ceramic discs (*n* = 7) had a thickness of 1.5 mm. With a D65 light source, TP values of 3.6 ± 1.5 were obtained for shade A2, and values of 4.0 ± 1.5 were obtained for shade A3. These results are consistent with those of the present study, in which TP values from 3.6 to 5.7 were obtained for one-step samples.

Translucency has been evaluated for different dental ceramic systems with specimens with a wide interval of thicknesses, as explained before. For feldspathic porcelains, a wide interval between 0.3 mm and 2.3 mm [3, 22, 23, 50] has been found. Although these values may be not clinically relevant, they show very interesting trends.

Wang et al. [27] studied the dependence of translucency on the thicknesses of dental porcelains. TP values ranged from 2.2 to 25.3, and translucency was inversely related to the thickness of the sample (i.e., translucency increased as the thickness decreased). For a thickness of 1.4 mm, TP varied from 3.5 to 17, and for a thickness of 1.6 mm, TP varied from 4.5 to 16. These results are consistent with those of the present study, where TP values from 1.65 to 5.7 were obtained with 1.5 mm thick discs using two ceramic paste preparation methods [27].

Lim et al. [18] measured the translucency of porcelain coatings and cores using a spectrophotometer and a spectroradiometer, and correlations between TP values were evaluated. The final thickness of porcelain discs was 1.5 mm, and TP values ranged from 4.4 to 12.5. There were significant differences in TP values obtained from each of the measuring instruments, although the results were highly correlated. Values obtained in that study were similar to those obtained in the present study.

The main limitations of the present *in vitro* study were that only one body/dentin porcelain, a single thickness, and a unique polishing protocol were used. The thickness (1.5 mm) used in this project is not clinic realistic because ceramic veneers and other ceramics/metal combinations are much thinner, but such thickness is currently used for all-ceramics restorations. Further studies should evaluate the effects of mixing preparation methods on translucency, color, and surface texture of different ceramic systems and thicknesses. In addition, more clinically oriented approaches, including the use of tooth geometries and technologies, such as spectrophotometers coupled to 3D scanners to evaluate *L∗a∗b∗* parameters directly, are suggested, and once such technologies become more massively available.

## 5. Conclusions

Considering the limitations of this *in vitro* study, the results showed that samples of the porcelain paste prepared with the incremental mixing method presented lower TP values than samples prepared with the one-step method, indicating that porcelain produced with the incremental mixing method is less translucent and rougher than the one produced with the one-step mixing method.

## Figures and Tables

**Table 1:** Highest probability intervals for the incremental and one-step mixing methods.

	Ceramic mixing	Method (95%)	Lower limit	Upper limit
Translucency parameter	Incremental	Bayesian	2.21	2.70
	One-step	Bayesian	4.26	4.73
	Incremental	Frequentist	2.28	2.60
	One-step	Frequentist	4.24	4.72

## Data Availability

Data are available upon request.
